# Composing drug delivery with light distribution improvement: the use of dissolving microneedles in skin cancer with photodynamic therapy

**DOI:** 10.1117/1.JBO.31.5.058001

**Published:** 2026-05-09

**Authors:** Michelle B. Requena, Cynthia E. Anderson, Dianeth S. Lima Bejar, Vladislav V. Yakovlev, Vanderlei S. Bagnato

**Affiliations:** a Texas A&M University, Department of Biomedical Engineering, College Station, Texas, United States; b University of Sao Paulo, Sao Carlos Institute of Physics, Sao Paulo, Brazil; c Texas A&M University, Artie McFerrin Department of Chemical Engineering, College Station, Texas, United States

**Keywords:** dissolving microneedles, photodynamic therapy, light, drug delivery, and skin cancer treatment

## Abstract

**Significance:**

Dissolving microneedles (MN) have emerged as a promising platform for drug delivery, providing a minimally invasive approach to bypass the skin’s natural barriers and enhance molecular penetration and diffusion. Their biocompatibility, user-friendly application, and ability to deliver precise therapeutic dosing make them particularly suitable for dermatological use. In addition to pharmacological benefits, dissolving MN possesses a geometric structure that enables optical waveguiding, thereby improving light penetration and distribution.

**Aim:**

We address a key limitation of photodynamic therapy (PDT): the limited penetration of light into biological tissues. PDT relies on activating photosensitizing agents with specific wavelengths of light to generate cytotoxic species, selectively targeting abnormal or diseased cells while minimizing effects on surrounding healthy tissue.

**Approach:**

Pyramidal dissolving MN arrays were fabricated from a biocompatible polymer and systematically characterized. Their light distribution profile under laser illumination was evaluated using image analysis.

**Results:**

Quantitative analysis of light distribution demonstrates that MN can simultaneously facilitate drug delivery and light distribution.

**Conclusions:**

This multifunctionality provides a synergistic therapeutic advantage, as localized drug release is complemented by optimized light delivery, thereby enhancing treatment outcomes. The dual-function platform has significant implications for PDT, enabling the design of integrated therapeutic systems that combine chemical and photonic modalities within a single, biodegradable device. Such systems may be particularly advantageous in resource-limited settings or outpatient care, where ease of use and effectiveness are essential. This strategy offers an approach to overcoming the limitations of conventional light-based therapies, supporting the development of more effective and accessible treatments for skin cancer and other dermatological conditions.

## 1 Introduction

**Fig. 1:**
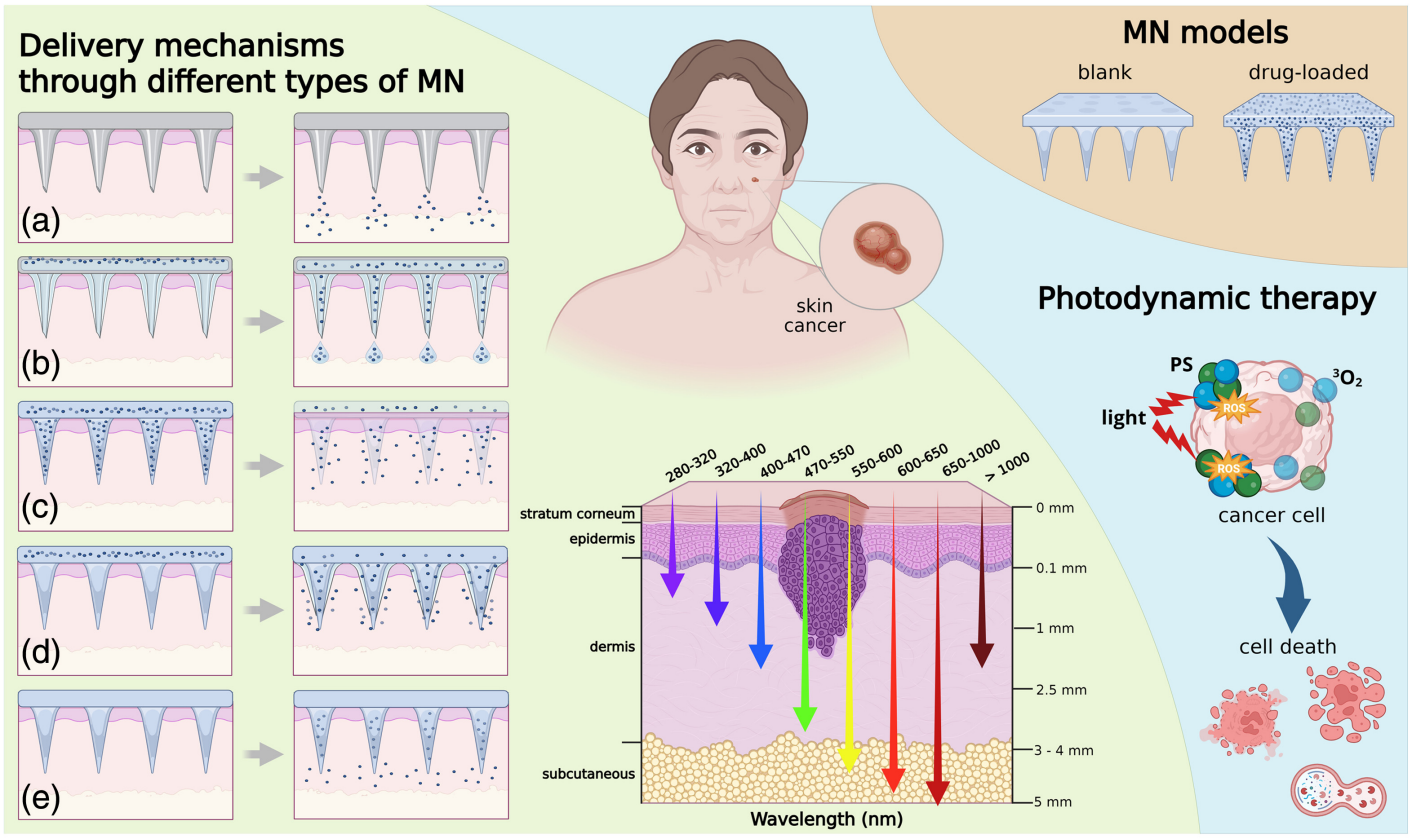
Schematic representation of MN technologies for transdermal drug delivery and optical guidance in PDT. Panels (a–e) illustrate five distinct MN designs: (a) solid MN that create microchannels for passive drug diffusion; (b) hollow MN that, after slight pressure or via a pump system, drug reservoirs are transported through the hollow channels; (c) dissolving MN composed of biodegradable polymers that dissolve and release drugs; (d) hydrogel-forming MN that swell upon contact with interstitial fluid, enabling sustained drug release; and (e) bioresponsive MN which, upon detection of the specific stimulus, the loaded drug is released in a controlled manner, allowing for precise, personalized therapy.^8^ The central diagram depicts skin architecture and the penetration depths of light at various wavelengths, from UV to near-infrared. The wavelength–depth relationship illustrated is schematic and tissue-composition dependent, with maximal light penetration occurring within the biological optical window (∼650 to 950 nm), where absorption by both endogenous chromophores and water is minimized. For wavelengths greater than 1000 nm, water absorption dominates, significantly reducing penetration depth. The right panel illustrates the application of MN in PDT, where light activation of a PS induces cytotoxic effects in cancer cells, targeting BCC with either drug-loaded or blank MN. Created in BioRender.^9^

Microneedles (MN) are minimally invasive devices that have been extensively investigated for transdermal drug delivery, as they can overcome the anatomical and physiological barriers of the skin.^1^^–^^4^ Polymeric MN facilitate the delivery of a variety of therapeutic agents, including anesthetics, antibiotics,^5^ drugs for chronic diseases,^6^ and nonsteroidal anti-inflammatory drugs,^7^ by creating microchannels that allow substances to reach deeper layers of the skin. MN technologies encompass several distinct designs:^2^^,^^3^ solid MN, which enable passive drug diffusion; hollow MN, which deliver drugs under pressure or via a pump system; dissolving MN, composed of biodegradable polymers that release their cargo as they dissolve; hydrogel-forming MN, which swell in response to interstitial fluid and provide sustained drug release; and bioresponsive MN, which release drugs in response to specific stimuli.^4^ These devices offer controlled, localized, and virtually painless administration. Figure 1 illustrates the major MN architectures and their role in transdermal drug delivery. Furthermore, MN provide a precise and reproducible means for delivering therapeutic agents across a broad spectrum of clinical applications.

MN devices have potential applications beyond drug delivery, such as facilitating the guidance and distribution of light into deeper tissues during light-based therapies.^10^^–^^13^ Critical design parameters such as geometry, tip shape, intertip spacing, height, material composition, and dissolution time directly influence drug release kinetics, mechanical properties, penetration depth, and cellular targeting.^10^ The dual functionality of drug dissolution and optical guidance in a single MN device synergistically enhances light penetration and therapeutic agent delivery to target lesions.

Photodynamic therapy (PDT) is a clinically established modality for treating malignant and premalignant superficial skin lesions, including actinic keratosis, squamous cell carcinoma in situ, and basal cell carcinoma (BCC).^14^^–^^16^ PDT is based on the activation of a photosensitizer (PS) by light in the presence of oxygen, leading to the generation of reactive oxygen species (ROS) and subsequent cell death.^17^^,^^18^ Despite its efficacy for superficial lesions, PDT is limited by insufficient light penetration into deeper tissue and inadequate delivery of topically applied PS precursors. Both the drug and light must reach the target tissue simultaneously at therapeutic doses. Still, red light (630 to 690 nm), although possessing greater tissue penetration, typically reaches a maximum depth of ∼4 to 5 mm,^19^ with the effective therapeutic dose restricted to about 2 mm due to absorption and scattering.^20^^,^^21^ This limitation restricts PDT to superficial lesions and increases the risk of incomplete treatment and lesion recurrence. Structural characteristics of tissue, such as high cellular density, poor vascularization, or keratinized layers, may further compromise light and drug penetration. Overcoming these intrinsic tissue barriers could significantly expand the therapeutic efficacy and clinical applicability of PDT in the management of skin lesions.

The penetration of topical PS, protoporphyrin IX (PpIX) precursors, including aminolevulinic acid (ALA) and methyl aminolevulinate (MAL), is also constrained, being influenced by lesion thickness, skin hydration, occlusion duration, and the presence of crusts or keratin. Alternative techniques, such as microneedling, exfoliation, or lipophilic carriers, have been explored to increase drug absorption,^22^^–^^29^ but these methods often introduce procedural complexity and variability in therapeutic outcomes.

MN address the principal limitations of PDT by enabling both controlled drug delivery and improved light guidance into deeper skin layers. This dual functionality has the potential to enhance therapeutic effects for thicker lesions, reduce recurrence rates, and expand the clinical indications for PDT. The design of MN can be optimized to achieve both pharmacological and optical objectives within a single platform, eliminating the need for additional devices such as lenses, fiber optics, or specialized light sources.

**Fig. 2:**
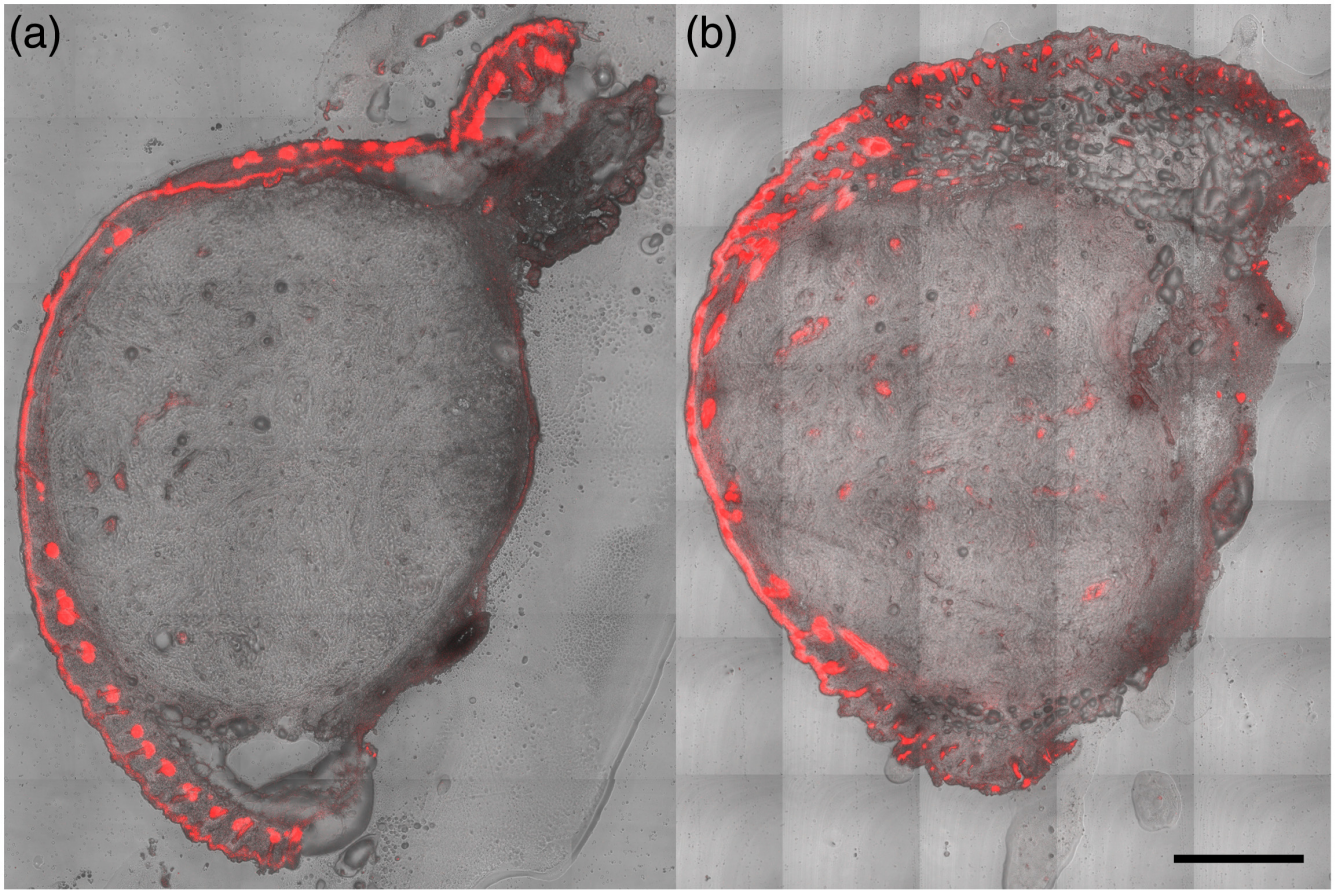
Confocal fluorescence microscopy images comparing PpIX (red fluorescence) accumulation in nonmelanoma tumor tissue from mice 2 h following administration of ALA via (a) topical cream and (b) dissolving MN. Scale bar: 500  μm.

In previous work, we have demonstrated that dissolving MN loaded with ALA provided superior therapeutic efficacy compared with ALA cream formulation in a murine tumor model.^30^^,^^31^ Figure 2 illustrates the advantage in terms of PpIX production and uniformity.

The images from previous studies^31^ highlight marked differences in PpIX localization and intensity. Following cream application [Fig. 2(a)], PpIX fluorescence is predominantly confined to the tumor epidermis and hair follicles. This pattern suggests limited and uneven ALA penetration. By contrast, MN-mediated delivery [Fig. 2(b)] results in a more uniform and deeper distribution of the PpIX signal throughout the entire tumor section. This finding indicates enhanced and homogeneous ALA uptake. These results demonstrate the superior ability of dissolving MNs to facilitate intratumoral delivery of ALA and promote widespread PpIX synthesis. This supports improved therapeutic efficacy of MN.

Building on these results, this study evaluates the light-propagation properties of previously used pyramidal MN *in vivo*.^30^ We describe the fabrication of these MN from a biodegradable polymer and comprehensively characterize their optical properties to confirm their efficacy in guiding and distributing light. We also address practical issues, procedural simplification, and patient adherence. Our findings highlight MN as an innovative dermatological therapy, particularly for cases where conventional PDT is limited by poor drug delivery or insufficient light penetration.

## 2 Material and Methods

### 2.1 Microneedles

**Fig. 3:**
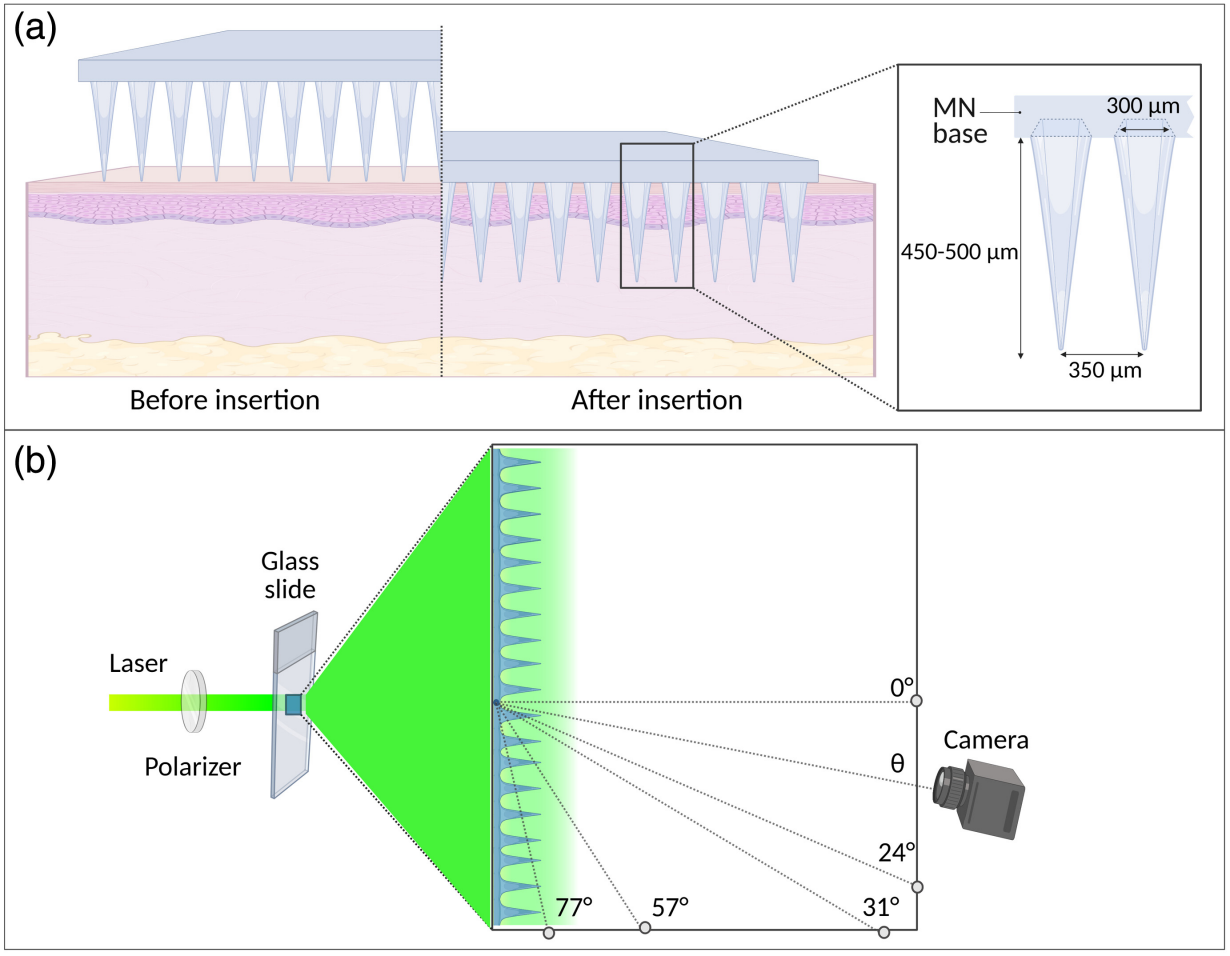
Schematic representation of the MN array and experiment setup: (a) MN penetrates the stratum corneum and reaches the dermis layer, bypassing the epidermal barrier after insertion. The magnified view shows individual MN dimensions, with a base diameter of 300  μm, intertip spacing of 350  μm, and tip lengths ranging from 450 to 500  μm; (b) Diagram showing the experimental arrangements used for the optical measurements. Created in BioRender.^9^

The mold utilized is an adaptation of a commercial flat mold from BlueAcre Technology company (Dundalk, Ireland). It features 19×19 square-based pyramidal holes; the MN array produced has tips measuring 450 to 500  μm in height and a base of 300  μm, with tips spaced 50  μm apart, as illustrated in Fig. 3. The polymeric base, sold commercially under the generic trademark Gantrez (Ashland, US), is a copolymer of methyl vinyl ether and maleic anhydride. The biodegradable molecules exhibit low toxicity, high biocompatibility, and bioadhesive properties.^32^^,^^33^ The base containing 30% w/w Gantrez AN-139 in water was prepared according to previously established protocols in the literature.^34^^,^^35^ The formulation containing 20% w/w polymer was then placed into the molds and then centrifuged (5702, Eppendorf, US) at 3500 rpm for 15 min. The MN arrays, which did not contain any drugs, were left to dry for 24 h at room temperature.

### 2.2 Irradiation Setup

To evaluate light propagation properties through the array, a Class 3B He-Ne laser (model 05-LGP-193, Carlsbad, CA, USA) with a wavelength of 543.5 nm was used as the light source. A polarizer was placed in the beam path to attenuate its power, which had a maximum output of 5 mW. Then, an MN array was attached to a glass slide. An RGB camera (HY-5299, ShenZhen Hayear Electronics Co., Ltd., China) was used to image the light profile passing through the array. The MN array was fixed and aligned with both the camera and the laser beam, thereby designating it as the initial position. Subsequently, lateral images were collected at four random angles (24 deg, 31 deg, 57 deg, and 77 deg), as illustrated in Fig. 3.

### 2.3 Image Processing

ImageJ (Free Software, NIH, USA)^36^ was used to extract pixel intensities from the green channel of 8-bit RGB images (0 to 255). Five random horizontal lines were manually drawn for each collected image to analyze the light intensity between the tips (labeled as “line”), and an additional five lines were drawn precisely at the tips (labeled as “MN”), as represented in Fig. 4. The mean intensity at the green channel as a function of distance was estimated to generate the light distribution profile across the array.

**Fig. 4:**
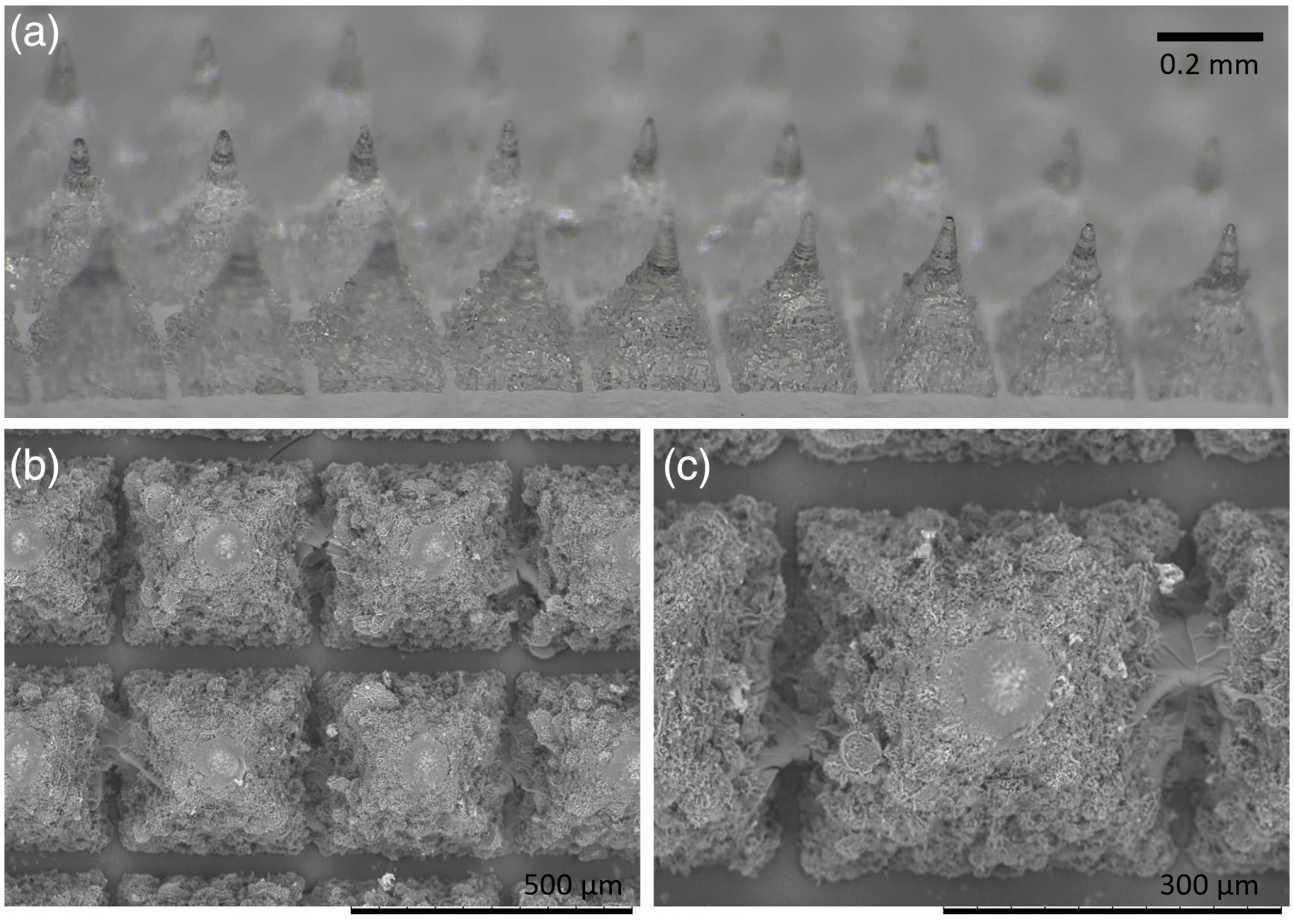
Morphological characterization of the MN array fabricated from 20% Gantrez AN-139; (a) array side-view optical image showing the uniform geometry and vertical alignment MN (scale bar: 0.2 mm), (b) top-view scanning electron microscopy (SEM; Hitachi TM300) image of the MN array, demonstrating the regular distribution and defined square bases of the structures (scale bar: 500  μm), and (c) magnified SEM image of a single MN base, revealing surface features and microstructural details resulting from the polymerization and molding process (scale bar: 300  μm).

## 3 Results and Discussions

Figure 4 presents microscopic images of MN morphology. Due to the mold fabrication method, the tips exhibit noticeable surface roughness. This is a common characteristic of MN production and, in this case, may even be advantageous by enhancing light scattering and distribution, as a rough surface naturally increases scattering compared with a smooth surface.

**Fig. 5:**
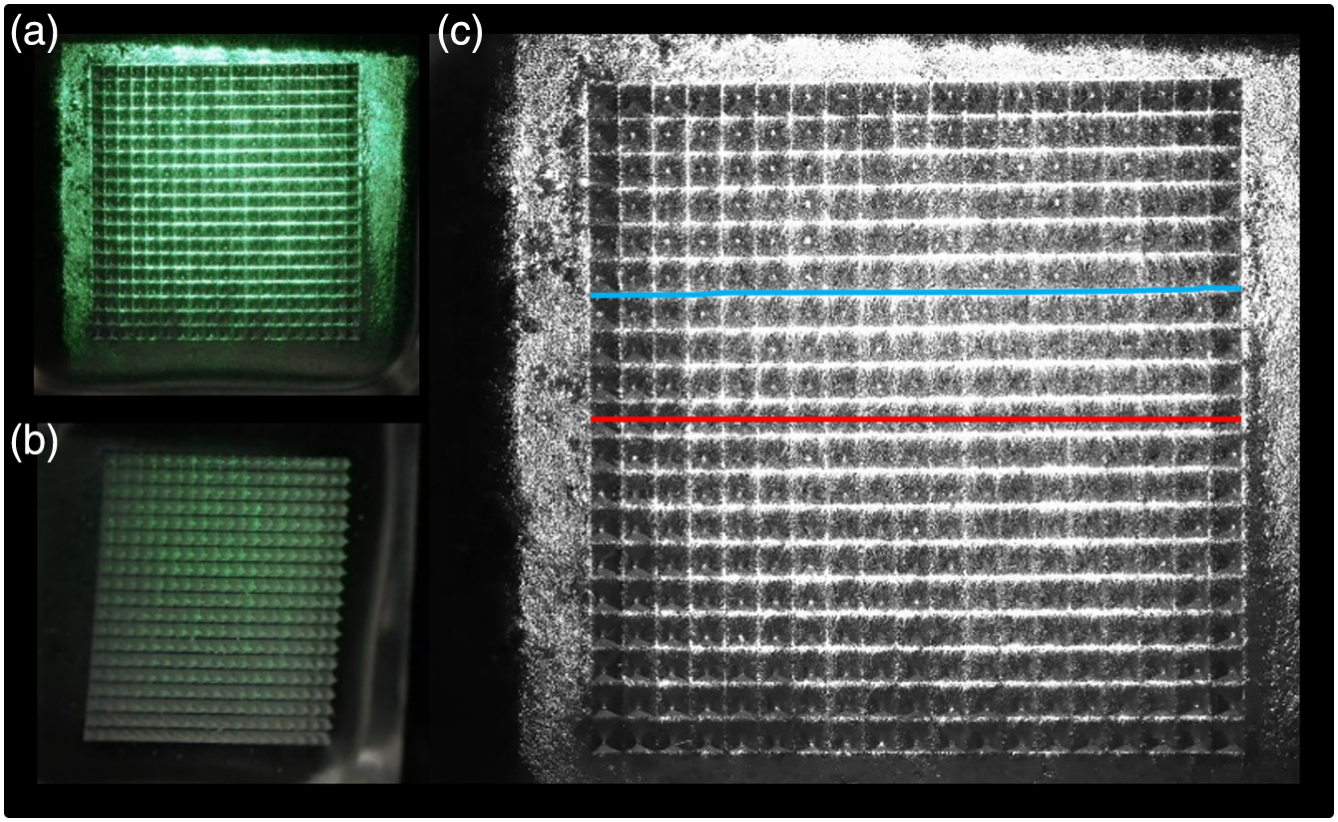
Optical assessment of light propagation through MN arrays at different camera angles relative to the laser beam direction. Panels (a) and (b) display the MN array illuminated by the laser and imaged at camera positions of (a) 0 deg (aligned with the incident beam) and (b) 57 deg (oblique angle). Panel (c) shows a processed image corresponding to the green channel extracted from the original RGB photograph, enhancing the contrast of the laser propagation through the array. The blue line indicates a region between MN tips, whereas the red line highlights a path passing through the centers of the MN tips.

Figure 5(a) displays images of light propagation through the MN array acquired at two different camera angles relative to the laser beam: 0 deg (top) and 57 deg (bottom). Figure 5(b) shows the corresponding processed images from the green channel of the original RGB photographs used for intensity quantification. The images collected at 77 deg were excluded from the analysis because, at this angle, the projection of the MN array made it difficult to accurately distinguish both the tips’ locations and the spaces between them, potentially compromising measurement accuracy.

These images show a clear difference in light propagation with respect to the angles of observation. In the initial position, the light is most intense between the tips, coinciding with their spacing, and is essentially a flat polymer layer with little to no light deviation. This high intensity is the same as that observed in the lateral of the array that corresponds to the film formed between the holes of the mold that will give origin to the pyramidal needles. As the angle relative to the initial position increases, light is seen more at the tips and with lower intensity in the spacing. The spacing transmitted the laser light in the same direction as its incidence, whereas the light must be deviated at the tips. Some parts of the incident light encounter the pyramid’s faces and undergo multiple internal reflections within the pyramidal structures. Due to multiple scattering events and imperfections in MN, light escapes in random directions, resulting in an almost isotropic distribution that propagates mainly forward, filling the space around and in front of the MN.

**Fig. 6:**
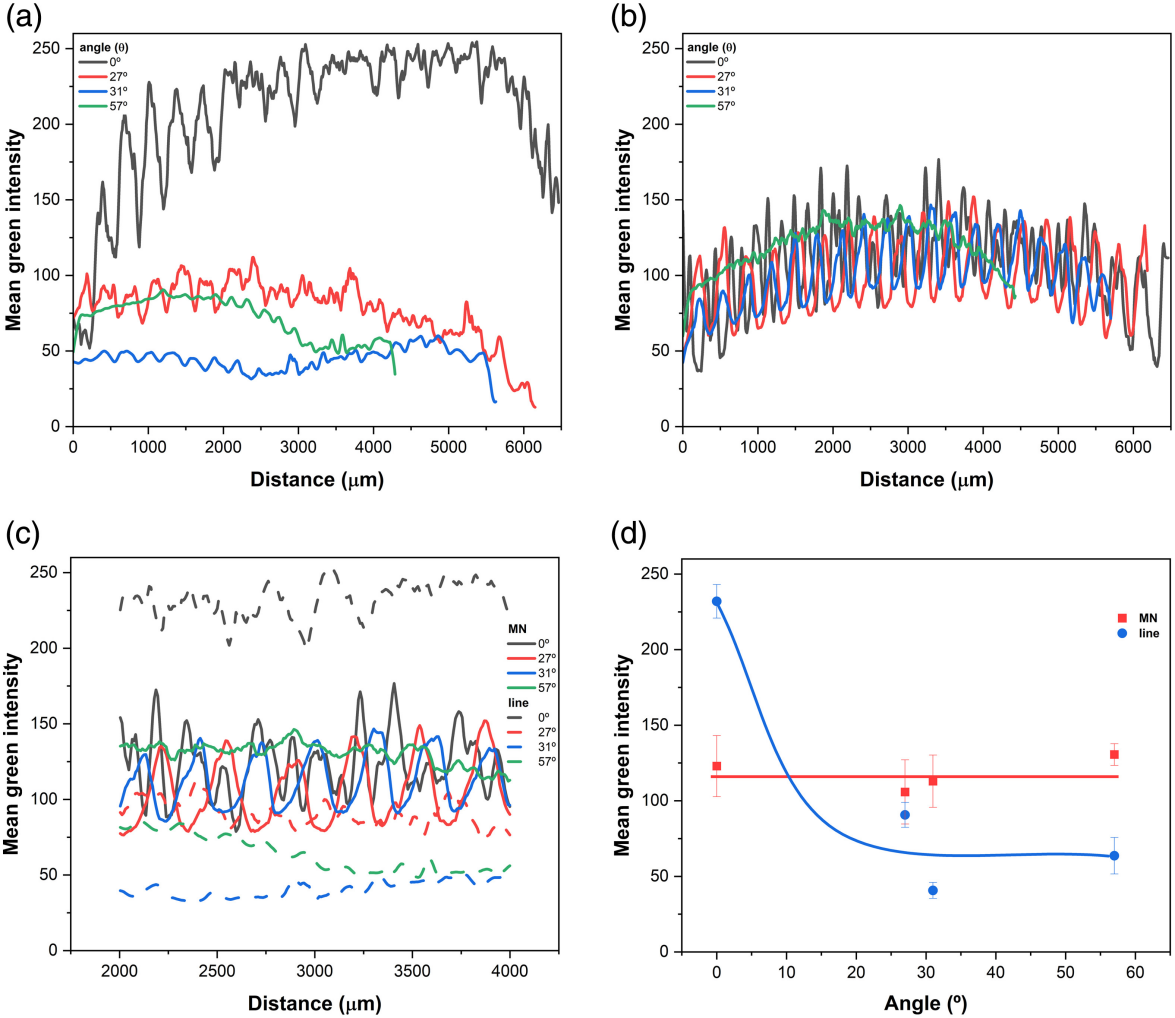
Light intensity analysis based on the green channel of RGB images, measured across different angles between the camera and the laser beam: (a) intensity variation between MN tips (“line”) and (b) directly on MN tips (“MN”); (c) light intensity profiles on both line and MN, considering the central laser exposure region (2000 to 4000  μm), across all angles; (d) angular dependence of the averaged light intensity for both “line” and “MN” regions.

The mean collected intensity as a function of the angle after light passes through the array is presented in Fig. 6. Although the overall light intensity depends on the distance after light passes through the array, the 0-deg collected light is shown to be more intense as a function of the distance because it corresponds to the incident transmitted light without deviation. As observed from different angles, the light that passes through the space between the tips without deviation decreases considerably. On the contrary, the light collected from the tips at different angles shows excellent uniformity with distance, indicating a high degree of isotropy in the light scattered by the MN. These results confirm that the MN array effectively functions as an isotropic emitter, facilitating uniform light distribution within the tissue.

Analysis of the intensity measurements between the tips showed that the maximum intensity was recorded at 0 deg, consistent with the expected theoretical model of light propagation. The intensity profiles exhibited minimal deviation at other angles, indicating a uniform light distribution. The intensity curves obtained at the MN tips further validate the isotropic behavior, as shown in Fig. 6(b). It showed that light intensity remains relatively invariant across various angles. This supports the conclusion that an isotropic light distribution is achieved throughout the MN array, indicating the potential to improve PDT using MN by promoting more homogeneous light distribution within tumors.

Figure 6(c) shows the light intensity as a function of angle, measured both in the spacing between the microneedles (labeled “lines”) and directly on the MN tips (labeled “MN”). The corresponding average intensity curves for both line and MN regions, across the same angular range, are presented in Fig. 6(d).

Although the intensity transmitted through the MN spacing (line) decreases considerably with viewing angle, the light emerging from the MN regions shows less angle dependence, with significant intensity in all directions. The importance of this effect introduced by MN in biomedical contexts, including PDT, is evident. Efficient delivery of light into complex, highly dispersive optical media, such as biological tissues, remains a fundamental technical challenge. Skin, for example, is a naturally turbid medium, composed of multiple layers with different refractive, scattering, and absorption properties. Under these conditions, the type of illumination adopted, isotropic or directed, directly impacts the uniformity of energy and depth distribution and, therefore, the efficacy of the treatment. Directed light (e.g., from lasers or focused LEDs) tends to follow a linear path, penetrating deeply into specific tissue regions and diffusing as it propagates. In turbid media, however, this light undergoes strong scattering and absorption, leading to rapid attenuation with depth. As a result, light energy is distributed unevenly, with superficial regions receiving high light doses, whereas deeper or lateral areas remain underexposed. This heterogeneous distribution compromises uniform PS activation during PDT, potentially resulting in incomplete coverage within treatment zones. On the contrary, isotropic light emitted in all directions from a diffusing source offers a more effective approach to homogeneously illuminating tissues, especially in cavities, thick lesions, or hard-to-reach areas. In turbid media, the diffuse nature of isotropic light enables better coupling with the tissue’s internal scattering, resulting in a more balanced volumetric distribution of optical energy, as discussed below.

**Fig. 7:**
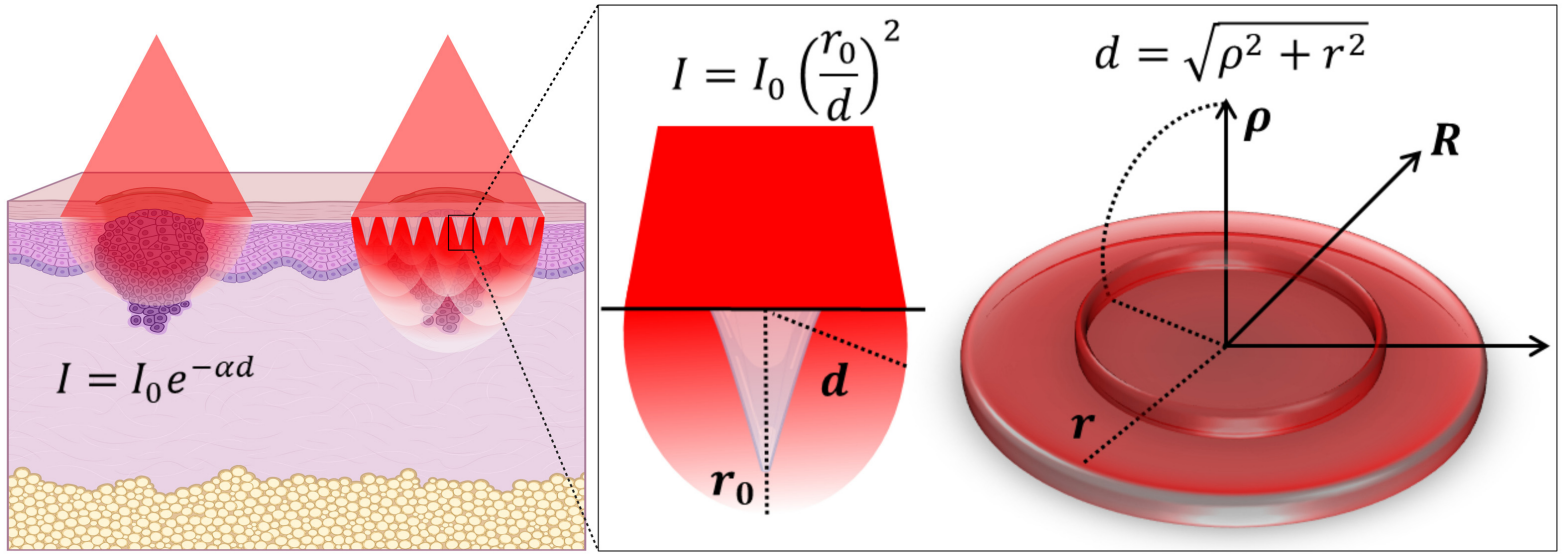
Schematics representing light propagation in a basal cell carcinoma. The left panel shows illumination directly in the tumor or through an MN array, with the Beer-Lambert equation describing exponential attenuation and geometric dispersion of light intensity. The central diagram illustrates the geometric relationship of the intensity distribution from a single tip, which diffuses light in all directions. The right panel demonstrates the parameters for radial and axial distances used in the calculations.

The efficacy of PDT is determined not only by total light dose but also by its spatial distribution within the tissue. Directional surface illumination often results in rapid attenuation and uneven PS activation, potentially leading to incomplete treatment and tumor recurrence. The MN array, with multiple spatially distributed optical emitters (each MN tip), produces significant angular redistribution of light, as demonstrated by the measurements and following calculations (Fig. 7).

Considering that each MN is an isotropic emitter with a circular source area of radius R, with emitters uniformly distributed at a surface density σ (number of emitters per unit area). The intensity from a single emitter at a distance d from the observation point follows the inverse-square law, where $I_{0}$ is the reference intensity at distance $r_{0}$ near the surface.

$$I=I_{0}(\frac{r_{0}}{d}{)}^{2},$$

To determine the total intensity I(ρ) detected at a point located at a distance ρ from the center of the circular source, we integrate the contributions from all emitters within the area. In polar coordinates, let r denote the radial distance from the center to an emitter within the source area, as shown in Fig. 7. Due to the symmetry of the circular source, the contributions from all emitters at a distance r are equivalent, leading to a simplified annular integration

$$d=\sqrt{\rho^{2}+r^{2}}.$$

The differential intensity contributed by an infinitesimal annular area at radius r is given by

$$dI=2\pi r\sigma I_{0}\frac{r_{0}^{2}}{\rho^{2}+r^{2}}dr.$$

Integrating over the entire source area, the total intensity at the observation point is

$$I(\rho )=\overset{R}{\underset{0}{\int }}2\pi \sigma I_{0}r_{0}^{2}\frac{r}{\rho^{2}+r^{2}}dr.$$

To resolve this integral, we substitute $x=r^{2}$, so that dx=2rdr

$$I(\rho )=\pi \sigma I_{0}r_{0}^{2}\overset{R^{2}}{\underset{0}{\int }}\frac{1}{\rho^{2}+x^{2}}dx=\pi \sigma I_{0}r_{0}^{2}\ln(\frac{\rho^{2}+R^{2}}{\rho^{2}}).$$

As absorption or attenuation is present in the medium, we introduce an attenuation coefficient α for any radial direction, and the intensity expression becomes

$$I(\rho )=\pi \sigma I_{0}r_{0}^{2}\ln(\frac{\rho^{2}+R^{2}}{\rho^{2}})e^{-\alpha \rho }.$$

This result demonstrates that the total intensity exhibits a logarithmic dependence on both the observation distance ρ and the radius R of the source area, further modulated by the emitter density and the attenuation characteristics of the medium. This means that when the light is isotropically distributed, the total intensity decaying along the distance from the surface is lower than that of directed light. In this context, the light scattering induced by the MN enables a broader, deeper light distribution, which is highly relevant to improving PDT outcomes.

To provide context, consider the comparison between an isotropic light source and one with direct incidence. The overall attenuation of intensity I decreased by ∼30% when direct light is coupled. Furthermore, if the illuminated surface area is extensive and only scattering processes are considered, the resulting intensity remains essentially constant over extended distances across the surface. These fundamental aspects of light propagation are crucial for designing systems that efficiently deliver optical energy in biomedical applications.

For instance, Coppola et al.^37^ investigated the optical properties of guiding and delivering light through optical microstructures made from poly(lactic-co-glycolic acid) (PLGA), a biodegradable copolymer, by varying the direction and shape of the MN tips. The arrays were placed on a glass slide, and a collimated light source was coupled to it and placed beneath. The light was focused through the MN’s base to the tip, and by adjusting the tip’s angle, it was guided by the tip. This preliminary study suggested that conical microstructures can also be light guides.

Similarly, Zhang et al.^12^ developed a device with LEDs emitting in the ultraviolet A region (UV-A, 360 nm) coupled to specific MN. Computational simulations and *in vitro* experimental results have also demonstrated the potential of MN arrays as waveguides, improving light delivery by up to 4 times for depths greater than 500  μm compared with conventional methods.

Champeau et al.^38^ described dissolving MN arrays loaded with ALA and illuminating them with an LED panel (DotStar, 630 nm), enabling uniform surface irradiation. Their protocol ($3.3\;\mathrm{mW}/cm^{2}$ for 90 min, in fractionated intervals) was developed to optimize photodynamic dose delivery while controlling heat buildup.

Beyond tip geometry, waveguide design further influences propagation and attenuation. A hydrogel planar waveguide model integrated with lens-MN is described as an optimal approach for delivering light to deep tissue. These lens-MN are designed to focus the incoming light beam into each MN at an optimal converging angle, reducing insertion loss and significantly increasing the light’s propagation length. Li et al.^13^ claimed that this device can effectively deliver light deep into solid tumors, including those in the lungs, breasts, or liver. However, the geometry of these devices can make attachment to *in vivo* skin tumors and delivery of high PDT doses challenging.

The generation of isotropic emissions within tissue also provides distinct advantages. Consider a scenario where a disk of light emission contains a specific density of emitters per unit area. These emission points of laser irradiation could emit energy uniformly or in a directed manner. When emerging from emitters with a directed light beam, scattering increases along the insertion depth, whereas absorption plays a less significant role, and the total intensity decays exponentially.

Although tissue scattering and absorption are reduced at red wavelengths^39^^,^^40^ commonly used in clinical PDT, the MN-induced angular redistribution of light is expected to remain effective due to the array’s geometric features and refractive index. This suggests that the advantages of isotropic emission observed in our models may translate to a range of clinically relevant spectral conditions.

The optical measurements were performed in a simplified glass-slide setup that does not fully capture the complexity of light behavior in living tissue. The experiments were useful for isolating the effects of MN geometry; real biological tissue could significantly alter the light redistribution effects we observed in air. Further work using tissue phantoms, *ex vivo* models, or computational simulations is needed to evaluate the effects of MN under more realistic conditions fully.

MN can be employed for PDT in two distinct strategies for combined drug delivery and light guidance. The first approach involves the sequential use of two separate arrays: an initial application of dissolving, drug-loaded MN (e.g., ALA-loaded MN) that facilitates intradermal delivery, followed by a second, nonloaded MN array designed exclusively to optimize irradiation. This strategy leverages the incubation period required for PpIX accumulation, thereby enabling optimized PS activation during the subsequent light-exposure phase. In this configuration, drug delivery and light administration are treated as independent yet complementary processes. In our PDT protocols for BCC treatment, irradiation is typically performed for 20 min at an irradiance of 125 mW/cm², corresponding to a total dose of $150\;J/{\mathrm{cm}}^{2}$.^22^^,^^41^^–^^45^ According to our previous preclinical results, this interval is insufficient to induce significant dissolution, thereby ensuring that the light distribution would remain consistent with the model described in this study. We demonstrated that MN containing 5% ALA exhibited minimal dissolution even after 1 h. To facilitate *in vivo* dissolution, a superficial heating protocol was required.^30^ Furthermore, when we tested a conical MN system loaded with 10% ALA, we observed that the MN remained largely intact for up to 2 h after insertion in the absence of external heating, despite evident PpIX formation.^31^ Thus, our findings indicate that the limited dissolution observed in our model is unlikely to impact the irradiation protocol, as the duration of light exposure remains within a timeframe in which significant MN dissolution does not occur. Alternatively, a single MN array can be used to enable both drug delivery and light application. As the MN tips dissolve primarily at their distal ends during the initial period, an incubation phase naturally occurs, after which irradiation can begin, either continuously or fractionated. This integrated approach eliminates the prolonged delay between PS administration and light exposure, allowing activation to occur as the drug reaches the target cells. Consequently, simultaneous use minimizes the risk that the drug diffuses into healthy tissues before activation. In this case, phototoxicity is more localized, preserving adjacent structures and increasing treatment selectivity. Synchronizing the application of light with the drug allows more precise control over where and when the therapeutic effect occurs. This is especially useful in irregular or difficult-to-access lesions, where exposure must be highly targeted. Eliminating excessive incubation time, where possible, speeds up treatment and makes it more efficient, reducing the patient’s time in the clinic and improving overall comfort during the procedure.^46^ In addition, with more controlled and localized activation, it is possible to minimize adverse reactions, such as pain, extensive inflammation, and light hypersensitivity following the procedure. Studies indicate that synchronization between the presence of the active PS and continuous illumination can increase ROS production, promoting greater selective damage to tumor cells.

Ultimately, we can conclude that the integrated or coordinated delivery of drugs and light in PDT marks a substantial advancement in skin cancer treatment. This strategy not only enhances clinical efficacy but also improves the overall patient experience by enabling greater precision, faster treatment times, and increased safety.

## 4 Conclusion

This study further validates the use of pyramidal dissolving MN as an efficient light-guiding device to enhance PDT outcomes. Simple optical measurements confirmed that this MN geometry induces multiple internal reflections, redistributing light angularly to produce an isotropic emission pattern. This isotropic distribution can significantly improve the uniformity of light within tissue, an essential factor for maximizing PDT efficacy, especially in turbid biological media such as skin, where conventional directional light sources often fail to achieve homogeneous and deep irradiation. Overall, this approach constitutes a meaningful advancement in PDT technology, offering a minimally invasive, targeted, and more efficient treatment modality. Future research should prioritize evaluating relevant preclinical models.

## Data Availability

The data that support the findings of this study are available from the corresponding author upon request.

## References

[1] Karim Z. Karwa P. Hiremath S. R. R., “Polymeric microneedles for transdermal drug delivery—a review of recent studies,” J. Drug Deliv. Sci. Technol. 77, 103760 (2022). 10.1016/j.jddst.2022.103760

[r2] Kulkarni D. et al., “Polymeric microneedles: an emerging paradigm for advanced biomedical applications,” Sci. Pharm. 91(2), 27 (2023). 10.3390/scipharm91020027

[r3] Aldawood F. K. Andar A. Desai S., “A comprehensive review of microneedles: types, materials, processes, characterizations and applications,” Polymers 13(16), 2815 (2021). 10.3390/polym13162815 34451353 PMC8400269

[4] Qi Z. et al., “Smart responsive microneedles for controlled drug delivery,” Molecules 28(21), 7411 (2023). 10.3390/molecules28217411 37959830 PMC10649748

[5] González-Vázquez P. et al., “Transdermal delivery of gentamicin using dissolving microneedle arrays for potential treatment of neonatal sepsis,” J. Controlled Rel. 265, 30–40 (2017). 10.1016/j.jconrel.2017.07.032 PMC5736097 28754611

[6] Migdadi E. M. et al., “Hydrogel-forming microneedles enhance transdermal delivery of metformin hydrochloride,” J. Controlled Rel. 285, 142–151 (2018). 10.1016/j.jconrel.2018.07.009 PMC6141810 29990526

[7] Sadeqi A. et al., “Hard polymeric porous microneedles on stretchable substrate for transdermal drug delivery,” Sci. Rep. 12(1), 1853 (2022). SRCEC3 2045-2322 10.1038/s41598-022-05912-6 35115643 PMC8813900

[8] Bao L. et al., “Recent advances in porous microneedles: materials, fabrication, and transdermal applications,” Drug Deliv. Transl. Res. 12(2), 395–414 (2021). 10.1007/s13346-021-01045-x 34415566 PMC8724174

[9] Requena M., https://BioRender.com/wxb0two (2026).

[10] Ali M. et al., “Dissolvable polymer microneedles for drug delivery and diagnostics,” J. Controlled Rel. 347, 561–589 (2022). JCREEC 0168-3659 10.1016/j.jconrel.2022.04.043 35525331

[r11] Champeau M. et al., “A facile fabrication of dissolving microneedles containing 5-aminolevulinic acid,” Int. J. Pharm. 586, 119554 (2020). IJPHDE 0378-5173 32652182 10.1016/j.ijpharm.2020.119554

[r12] Zhang H. et al., “Biocompatible light guide-assisted wearable devices for enhanced UV light delivery in deep skin,” Adv. Funct. Mater. 31(23), 2100576 (2021). AFMDC6 1616-301X 10.1002/adfm.202100576

[13] Li L. P. et al., “Biocompatible and implantable hydrogel optical waveguide with lens-microneedles for enhancing light delivery in photodynamic therapy,” Adv. Photonics Res. 5(8), 2400031 (2024). 10.1002/adpr.202400031

[14] Trakatelli M. et al., “Update of the European guidelines for basal cell carcinoma management: developed by the guideline subcommittee of the European Dermatology Forum,” Eur. J. Dermatol. 24(3), 312–329 (2014). EJDEE4 1167-1122 10.1684/ejd.2014.2271 24723647

[r15] National Comprehensive Cancer Center, “NCCN clinical practice guidelines in oncology,” 2020, www.nccn.org

[16] Peris K. et al., “Diagnosis and treatment of basal cell carcinoma: European consensus–based interdisciplinary guidelines,” Eur. J. Cancer 118, 10–34 (2019). EJCAEL 0959-8049 10.1016/j.ejca.2019.06.003 31288208

[17] Wilson B. C., “Photodynamic therapy for cancer: principles,” Can. J. Gastroenterol. 16(6), 393–396 (2002). 10.1155/2002/743109 12096303

[18] Wilson B. C. Patterson M. S., “The physics, biophysics and technology of photodynamic therapy,” Phys. Med. Biol. 53(9), R61–R109 (2008). PHMBA7 0031-9155 10.1088/0031-9155/53/9/R01 18401068

[19] Ruggiero E. et al., “Upconverting nanoparticles for the near infrared photoactivation of transition metal complexes: new opportunities and challenges in medicinal inorganic photochemistry,” Dalton Trans. 45(33), 13012–13020 (2016). DTARAF 1477-9226 10.1039/C6DT01428C 27482656

[20] Ash C. et al., “Effect of wavelength and beam width on penetration in light-tissue interaction using computational methods,” Lasers Med. Sci. 32(8), 1909–1918 (2017). 10.1007/s10103-017-2317-4 28900751 PMC5653719

[21] Kwon K. et al., “Enhancement of light propagation depth in skin: cross-validation of mathematical modeling methods,” Lasers Med. Sci. 24(4), 605–615 (2009). 10.1007/s10103-008-0625-4 19030946

[22] Requena M. B. et al., “Intradermal delivery of methyl aminolevulinate by dermograph for BCC treatment using PDT: a randomized clinical trial,” Photodiagn. Photodyn. Ther. 41, 103446 (2023). 10.1016/j.pdpdt.2023.103446

[r23] Wiegell S. R., “Pain associated with photodynamic therapy using 5-aminolevulinic acid or 5-aminolevulinic acid methylester on tape-stripped normal skin,” Arch. Dermatol. 139(9), 1173 (2003). 10.1001/archderm.139.9.1173 12975159

[r24] Arora A. Prausnitz M. R. Mitragotri S., “Micro-scale devices for transdermal drug delivery,” Int. J. Pharm. 364(2), 227–236 (2008). IJPHDE 0378-5173 10.1016/j.ijpharm.2008.08.032 18805472 PMC2752650

[r25] Torezan L. et al., “A pilot split-face study comparing conventional methyl aminolevulinate- photodynamic therapy (PDT) with microneedling-assisted PDT on actinically damaged skin,” Dermatol. Surg. 39(8), pp. 1197–1201 (2013). 10.1111/dsu.12233 23638986

[r26] Petukhova T. A. et al., “Effect of expedited microneedle-assisted photodynamic therapy for field treatment of actinic keratoses: a randomized clinical trial,” JAMA Dermatol. 153(7), 637–643 (2017). 10.1001/jamadermatol.2017.0849 28514458 PMC5543325

[r27] Liao C. et al., “Photodiagnosis and photodynamic therapy combination curettage and modified ALA-PDT for multiple basal cell carcinomas of the face and head,” Photodiagn. Photodyn. Ther. 35(April 2021), 102393 (2023). 10.1016/j.pdpdt.2021.102393 34116251

[r28] Lopez R. F. V et al., “Enhanced delivery of 5-aminolevulinic acid esters by iontophoresis in vitro,” Photochem. Photobiol. 77(3), 304–308 (2003). PHCBAP 0031-8655 10.1562/0031-8655(2003)077<0304:EDOAAE>2.0.CO;2 12685659

[29] van den Akker J. T. H. M. et al., “Topical application of 5-aminolevulinic acid hexyl ester and 5-aminolevulinic acid to normal nude mouse skin: differences in protoporphyrin IX fluorescence kinetics and the role of the stratum corneum,” Photochem. Photobiol. 72(5), 681–689 (2000). PHCBAP 0031-8655 10.1562/0031-8655(2000)072<0681:TAOAAH>2.0.CO;2 11107855

[30] Requena M. B. et al., “Dissolving microneedles containing aminolevulinic acid improves protoporphyrin X distribution,” J. Biophotonics 14(1), jbio.202000128 (2021). 10.1002/jbio.202000128 32981235

[31] Bejar D. S. L. et al., “Scalable fabrication of polymeric dissolving microneedles for optimized ALA delivery in photodynamic therapy,” J. Photochem. Photobiol. B 274, 113319 (2026). JPPBEG 1011-1344 10.1016/j.jphotobiol.2025.113319 41352277

[32] Gardner C. M. et al., “Poly(methyl vinyl ether-alt-maleic acid) polymers for cell encapsulation,” J. Biomater. Sci. Polym. Ed. 22(16), 2127–45 (2011). JBSEEA 0920-5063 10.1163/092050610X535149 21067656

[33] Iglesias T. et al., “In vitro evaluation of the genotoxicity of poly(anhydride) nanoparticles designed for oral drug delivery,” Int. J. Pharm. (2017). IJPHDE 0378-5173 10.1016/j.ijpharm.2017.03.016 28286081

[34] Rzhevskiy A. S. et al., “Microneedles as the technique of drug delivery enhancement in diverse organs and tissues,” J. Controlled Rel. 104(10), 184–202 (2014). JCREEC 0168-3659 10.1016/j.jconrel.2017.11.048 29203415

[35] Raj Singh T. R. et al., “Investigation of swelling and network parameters of poly(ethylene glycol)-crosslinked poly(methyl vinyl ether-co-maleic acid) hydrogels,” Eur. Polym. J. 45(4), 1239–1249 (2009). EUPJAG 0014-3057 10.1016/j.eurpolymj.2008.12.019

[36] Schneider C. A. Rasband W. S. Eliceiri K. W., “NIH image to ImageJ: 25 years of image analysis,” Nat. Methods 9(7), 671–675 (2012).1548-7091 10.1038/nmeth.2089 22930834 PMC5554542

[37] Coppola S. et al., “Transmitting light through biocompatible and biodegradable drug delivery micro needles,” IEEE J. Sel. Top. Quantum Electron. 27(5), 1–8 (2021). IJSQEN 1077-260X 10.1109/JSTQE.2021.3057834

[38] Champeau M., Dissolving Microneedles for an Optimal Transdermal Delivery of an Active Principle Used in Photodynamic Therapy : Development and Proof of Concept, Université de Lille (2020).

[39] Zima N. G. et al., “Correlations of light scattering properties in human skin with the person’s age assessed using a non-invasive technique,” Biomed. Opt. Express 15(6), 3817–3830 (2024). BOEICL 2156-7085 10.1364/BOE.523183 38867783 PMC11166447

[40] Kono T. Yamada J., “In vivo measurement of optical properties of human skin for 450–800 nm and 950–1600 nm wavelengths,” Int. J. Thermophys. 40(5), 51 (2019). IJTHDY 0195-928X 10.1007/s10765-019-2515-3

[41] Ramirez D. P. et al., “Single visit PDT for basal cell carcinoma—a new therapeutic protocol,” Photodiagn. Photodyn. 26, 375–382 (2019). 10.1016/j.pdpdt.2019.04.016 31002888

[r42] Salvio A. G. et al., “The use of a portable device for photodynamic therapy at home decreasing the patient’s stay at hospital for small nodular basal cell carcinoma treatment,” Photodiagn. Photodyn. Ther. 41, 103449 (2023). 10.1016/j.pdpdt.2023.103449

[r43] Salvio A. G. et al., “Photodynamic therapy as a treatment option for multiple pigmented basal cell carcinoma: long-term follow-up results,” Photodiagn. Photodyn. Ther. 33, 102154 (2021). 10.1016/j.pdpdt.2020.102154 33348074

[r44] Salvio A. G. et al., “A new photodynamic therapy protocol for nodular basal cell carcinoma treatment: effectiveness and long-term follow-up,” Photodiagn. Photodyn. Ther. 37(July 2021), 1–7 (2022). 10.1016/j.pdpdt.2021.102668 34863948

[45] Salvio A. G. et al., “Effective treatment depth of photodynamic therapy after partial debulking of nodular basal cell carcinoma,” Int. J. Dermatol. 56, 104960 (2025). 10.1016/j.pdpdt.2025.104960 40205644

[46] Salvio A. G. et al., “Long-term follow-up results of a pilot study for nodular basal cell carcinoma with PDT using partial home treatment protocol,” Photodiagn. Photodyn. Ther. 45, 103930 (2024). 10.1016/j.pdpdt.2023.103930 38103584

